# Comparison of two viscoelastic testing devices in a porcine model of surgery, hemorrhage and resuscitation

**DOI:** 10.3389/fbioe.2024.1417847

**Published:** 2024-08-13

**Authors:** Daniel Gruneberg, Maximilian Dietrich, Alexander Studier-Fischer, Clara Petersen, Maik von der Forst, Berkin Özdemir, Herbert Schöchl, Felix Nickel, Markus A. Weigand, Felix C. F. Schmitt

**Affiliations:** ^1^ Department of Anesthesiology, Medical Faculty Heidelberg, Heidelberg University, Heidelberg, Germany; ^2^ Department of General, Visceral, and Trasplantation Surgery, Heidelberg University Hospital, Heidelberg, Germany; ^3^ Ludwig Boltzmann Institute for Traumatology, The Research Center in Cooperation with AUVA, Vienna, Austria

**Keywords:** viscoelastic hemostatic assays, device comparison, swine, coagulopathy, ClotPro^®^

## Abstract

**Introduction:**

Viscoelastic hemostatic assays (VHA) are integral in contemporary hemostatic resuscitation, offering insights into clot formation, firmness, and lysis for rapid diagnosis and targeted therapy. Large animal models, particularly swine, provide anatomical and physiological analogies for coagulation research. Despite the growing use of VHAs, the ClotPro^®^ device’s applicability in porcine models remains unexplored. This study investigates ClotPro^®^ in a porcine model of abdominal surgery, severe hemorrhage, and resuscitation, comparing it with the established ROTEM^®^ delta system.

**Methods:**

Twenty-seven healthy pigs underwent abdominal surgery, hemorrhage and resuscitation. ClotPro^®^ and ROTEM^®^ were used to assess viscoelastic hemostatic properties at baseline, after surgery, 60 min after shock induction, 60 and 120 min after resuscitation.

**Results:**

Clotting times in extrinsically and intrinsically stimulated assays exhibited fair to moderate correlation. Clot firmness in extrinsically stimulated tests could be used interchangeably while fibrin polymerization assays revealed significant differences between the devices. Fibrin polymerization assays in ClotPro^®^ consistently yielded higher values than ROTEM^®^. Furthermore, the study evaluated the ClotPro^®^ TPA-test’s applicability in porcine blood, revealing failure of lysis induction in porcine blood samples.

**Conclusion:**

This research contributes valuable insights into the use of ClotPro^®^ in porcine models of hemorrhage and coagulopathy, highlighting both its applicability and limitations in comparison to ROTEM^®^ delta. The observed differences, especially in fibrin polymerization assays, emphasize the importance of understanding device-specific characteristics when interpreting results. Due to its inapplicability, TPA-test should not be used in porcine blood to evaluate fibrinolytic potential. The study provides a foundation for future investigations into the use of different viscoelastic hemostatic assays in porcine animal models.

## 1 Introduction

Point of care viscoelastic hemostatic assays (VHA) play a crucial role in contemporary hemostatic resuscitation. They offer comprehensive insights in clot formation, firmness and lysis and therefore facilitate diagnosis and targeted therapy of coagulopathy (Zipperle et al., 2023). They can be performed directly at the patient’s bedside and therefore reduce preanalytical time span since transportation of samples to the laboratory facility could be omitted. Haas et al. (2012) found an time benefit of 11 min when analysis were made at the bedside. Beneficial effects of the use of VHA have been demonstrated for the need of blood product transfusion (Fahrendorff et al., 2017) and overall costs (cost for blood products, medication, coagulation analysis and unwanted events) (Kuiper et al., 2019). Due to their comprehensive evaluation of the coagulation process and swift turnover times, VHAs are well-established tools in clinical practice and research.

In light of ethical and technical considerations, large animal models are frequently employed in research investigating coagulopathy. Among various species, swine represent a pivotal animal model for coagulation and hemostatic resuscitation. Swine models of bleeding and hemorrhage are used in different fields from trauma (Brännström et al., 2021; McDonough et al., 2022) over surgery (Nguyen et al., 1998) to ECMO (Reed et al., 2020) and other types of hemorrhage (Tomori et al., 2010). In a review of large animal models of trauma and bleeding, Rayatdoost and Grottke (2023) found more than 70 studies using pigs as the experimental platform. Coagulopathy involves a complex interplay of cell-based hemostasis, plasmatic coagulation, vasculature, tissue and hemodynamic properties (Schochl et al., 2024). Large animal models like swine offer great analogy in anatomical and physiological circumstances (Ask et al., 2022; Rayatdoost and Grottke, 2023). They mimic (patho) physiologic conditions better then small animal or *ex vivo* models and therefore facilitate the transfer of research to clinical care.

Due to the aforementioned benefits, VHAs are used in many porcine models studying coagulopathy. Several studies reported the use of ROTEM^®^ and TEG^®^ in porcine animal models (Martini et al., 2022; Storm et al., 2022; Gerling et al., 2023). However, with the growing use of viscoelastic hemostatic testing, new devices have entered the market. One of these devices is the ClotPro^®^, which shares basic principles with ROTEM^®^ and TEG^®^ but exhibits slight technical differences. In contrast to ROTEM^®^, ClotPro^®^ uses active pipetting tips containing dry reagents and a fixed pin and rotating cup. Several clinical investigations examined the interchangeability of results obtained by the new ClotPro^®^ and established ROTEM^®^. They found that results cannot be used interchangeably in any case (Infanger et al., 2021; Yoshii et al., 2022; Gruneberg et al., 2024).

To date, there is no published data evaluating the use of ClotPro^®^ in porcine animal studies.

Therefore, we examined the applicability of ClotPro^®^ in porcine blood and the degree of interchangeability between ROTEM^®^ and ClotPro^®^ in swine. Further we determined the magnitude of differences in different ranges of measurement and evaluated the use of TPA-test which is exclusively available for the ClotPro^®^ device.

To answer these questions, we used an established porcine model of abdominal surgery, severe hemorrhage and resuscitation and measured viscoelastic hemostatic properties using ClotPro^®^ and ROTEM^®^ at five different timepoints over the course of experiment.

## 2 Materials and methods

The study was part of another research project which is described elsewhere 27 healthy pigs (*sus scrofa* domestica) were examined as described by Dietrich et al., 2021.

### 2.1 Ethical approval

The study was approved by the appropriate governmental body (Regierungspräsidium Karlsruhe, file reference G-261/19) and conducted in accordance with the European law on the protection of animals used for scientific purposes (EU-Directive 2010/63).

### 2.2 Animal handling and anesthesia

All pigs were kept at Interfaculty Biomedical Facility of the University of Heidelberg under constant room temperature and a fixed circadian rhythm. They fasted the day of surgery. Water was accessible *ad libitum* until anesthesia induction.

Anesthesia was induced by an intramuscular injection of 1 mg/kg midazolam (Midazolam-hameln^®^5 mg/mL by Hameln pharma plus GmbH^®^, Hameln, Germany) and 10 mg/kg ketamine (Ketamin 10%^®^ by Heinrich Fromme^®^, Warburg, Germany) to the animals neck.

A venous-catheter was placed at the animals’ ear after loss of consciousness. 2 mg/kg of propofol were administered before airway management. After anesthesia induction endotracheal intubation and mechanical ventilation were initiated. Maintenance of anesthesia was conducted by a combination of intravenous 0.5–1 mg/kg/h midazolam, 10–20 mg/kg/h ketamine, and inhalational Sevoflurane^®^ (exsp. vol% 1.5–2.5). Animals were equipped with a central venous line in the right internal jugular vein and an arterial line in the right femoral artery for hemodynamic measurements, shock induction and blood sampling.

### 2.3 Surgery

As an equivalent for a major abdominal surgical procedure, the pigs underwent esophagectomy according to Ivor Lewis. Further animals received an exposure of the right kidney which was necessary in context of another study which was performed in parallel in the same animals (Dietrich et al., 2021). The parallel-study used hyperspectral imaging for non-invasive evaluation of organ perfusion. It is not to be expected that these measurements had impact on any coagulation parameter or any measurement presented in this study.

### 2.4 Major hemorrhage

Induction of hemorrhage followed surgical procedure. Shock was induced by bloodletting via a central venous line placed within the right internal jugular vein. Blood was drawn till a target mean arterial pressure (MAP) of 40 ± 5 mmHg was achieved. If necessary further blood was taken to maintain the target MAP for 65 min.

### 2.5 Resuscitation

All animals received a basic crystalloid infusion of 10 mL/kg/h (Sterofundin ISO^®^ by B. Braun^®^, Melsungen, Germany). For intervention animals were randomized to one of three treatment arms. First one contained no intervention, the second comprise crystalloid resuscitation and the third one norepinephrine treatment. Resuscitation was performed to achieve a target MAP of 65 mmHg within the first hour and 90 mmHg for the second hour. To achieve the target-MAP the fluid group received further crystalloid infusion (Sterofundin^®^, B. Braun SE, Melsungen, Germany) while the norepinephrine-treated group received an intravenous infusion of norepinephrine (Arterenol^®^, Sanofi-Aventis Deutschland GmbH, Höchst, Germany). In the control group no hemodynamic resuscitation was performed.

### 2.6 Hemostatic measurements

Blood samples for viscoelastic hemostatic assays (VHA) were collected at five different timepoints: T0 = Baseline after anesthesia induction, T1 = after surgery, T2 = 60 min after hemorrhage, T3 and T4 = 60 and 120 min after start of resuscitative treatment respectively.

Blood samples for blood gas analysis were taken from femoral arterial line, all other blood samples were taken from central venous line. Citrate-anticoagulated whole blood was used for ROTEM^®^ and ClotPro^®^ analysis. All measurements were performed by the same experienced experimenter immediately after blood sampling. For ClotPro^®^ six channels were run (Ex-test, In-test, Fib-test, Ap-test, NA-test and TPA-test). Extrinsically stimulated tests (Ex-test/Ex-TEM) contain recombinant tissue factor as the initiating reagent of the coagulation process. These tests represent the extrinsic pathway of the coagulation cascade. Intrinsically stimulated tests (IN-test/IN-TEM) use ellagic acid as the initiating reagent. These tests represent the intrinsic pathway of the coagulation cascade.

Fibrin polymerization assays are based on extrinsically stimulated tests and contain platelet inhibitors to eliminate the platelet component from clot firmness. AP tests include aprotinin as an antifibrinolytic agent to detect hyperfibrinolysis by comparison of clot firmness with extrinsically stimulated tests. Fibrinolysis is stimulated by a recombinant plasminogen activator in the TPA test. NA tests are unstimulated tests where blood samples are recalcified without any additional stimulating agent. Since equivalents for NA-test and TPA-test are not available for ROTEM^®^, only four channels (EX-TEM, IN-TEM, FIB-TEM and AP-TEM) were performed there. ClotPro^®^ and ROTEM^®^ hemostatic assays were run for 60 min. All measurements were performed according to the respective manufacturers’ guidelines.

### 2.7 Euthanasia

Deeply anaesthetised pigs were euthanized with intravenous injection of potassium chloride. Death was confirmed by ECG and etCO2.

### 2.8 Statistical analysis

Statistical analyses were performed using SAS version 9.4 (SAS Institute Inc., Cary, NC, United States). Statistical measurements were performed as described elsewhere (Gruneberg et al., 2024). Comparison of the two VHA devices was accessed by fit plots and Bland-Altman plots. Correlations between ClotPro^®^ and ROTEM^®^ delta were calculated as Pearson’s correlations coefficient with corresponding 95% CI. The correlations were classified as poor (0.1–0.2), fair (0.3–0.5), moderate (0.6–0.7), very strong (0.8–0.9), and perfect (1.0) according to Chan, (2023). Thereby classification of correlation strength according to Chen et al. was meant as a rough guideline. Since correlation coefficient may be influenced by a small subset of datapoints, the degree of correlation should not be quantified only by magnitude of the r-value. For detailed evaluation of the relationships, scatter plots with all single data points were provided. For Bland-Altman plots, average value between both methods was plotted against the differences between the respective ClotPro^®^ and ROTEM^®^ parameters. Bland-Altman plots were presented as absolute and percentage values.

## 3 Results

### 3.1 Animals

A total of 27 pigs (weight 35.6 ± 2.6 kg) were assessed in the study. One animal died before the first measurements, another two animals died during the observation period between T2 and T3. Data obtained from animals which died over the course of experiment were included as far as possible. Finally, data from 26 pigs were eligible for analysis.

### 3.2 Framework conditions of hemostasis

Baseline conditions and changes due to shock and resuscitation are shown in Table 1. After anesthesia induction animals presented with normal temperature, hemoglobin, pH and lactate values. Under fluid-only resuscitation temperature dropped to 34.5°C after 120 min of intervention. In all other groups temperature was kept sufficient over the whole course of experiment. Fluid resuscitation also leads to a decrease in hemoglobin values while norepinephrine resuscitation caused significant lactate acidosis. Considering hemodynamics no single intervention was capable to achieve the targeted MAP values. Actual MAP values reached a maximum of 60 mmHg in the fluid group and 40 mmHg in the other two groups. A detailed illustration of hemodynamic changes over the course of experiment were published elsewhere by Dietrich et al. (2021).

**TABLE 1:** framework conditions of hemostasis by group and timepoint.

Baseline (after anesthesia)				P1	P2	P3
	Control	Fluid	catecholamine			
Temperature [°C]	36.9 ± 0.9	36.5 ± 0.8	36.4 ± 0.6	0.38	0.31	0.93
Hb [g/dL]	9.3 ± 0.8	9.9 ± 0.8	10.2 ± 1.8	0.37	0.19	0.65
pH	7.489 ± 0.04	7.489 ± 0.026	7.487 ± 0.051	0.99	0.94	0.92
Lactate [mmol/L)	1.98 ± 1.55	1.42 ± 0.31	1.55 ± 0.73	0.28	0.40	0.79
Fluid volume [ml]	353 ± 137	303 ± 222	204 ± 145	0.56	0.10	0.27

T2 (60 min shock)						
	Control	Fluid	catecholamine			
Temperature [°C]	35.9 ± 0.7	35.9 ± 1.2	35.9 ± 0.8	0.95	0.93	0.88
Hb [g/dL]	9.2 ± 1.1	9.7 ± 0.6	9.4 ± 0.5	0.26	0.69	0.42
pH	7.395 ± 0.024	7.433 ± 0.055	7.440 ± 0.038	0.10	0.05	0.76
Lactate [mmol/L)	2.06 ± 1.17	2.42 ± 0.47	2.21 ± 0.54	0.40	0.72	0.61
Fluid volume [ml]	2057 ± 647	1786 ± 250	1757 ± 377	0.25	0.20	0.90

T4 (120 min resuscitation)						
	Control	Fluid	catecholamine			
Temperature [°C]	35.9 ± 1.3	34.5 ± 1	36.7 ± 1.2	0.04	0.29	<0.01
Hb [g/dL]	8.2 ± 1.7	7.1 ± 0.9	9.9 ± 1.2	0.12	0.01	<0.01
pH	7.378 ± 0.029	7.407 ± 0.057	7.296 ± 0.053	0.25	<0.01	<0.01
Lactate [mmol/L)	3.18 ± 2.27	2.05 ± 0.59	6.94 ± 1.63	0.21	<0.01	<0.01
Fluid volume [ml]	2,967 ± 703	6,774 ± 1,176	2,726 ± 523	<0.01	0.57	<0.01

Temperature measured as °C; Hb, hemoglobin measured as g/dL; lactate measured as mmol/L; Fluid volume, cumulatively infused fluids measured in ml; Control, without resuscitation; fluid, crystalloid infusion; catecholamine, norepinephrine; all parameter were stated as mean ± standard deviation; *p*-values: P1, control vs fluid; P2, control vs catecholamine; P3, fluid vs catecholamine; normal ranges: Hb 9–10 g/dL; Lactate under resting conditions 0.5–2.0 mmol/L; pH 7.35–7.45; temperature: 38.3°C–39.4°C.

For device comparison data from all groups and timepoints were pooled to access device specific differences across a broad range of measurement and under different framework conditions.

### 3.3 Viscoelastic measurements at baseline, in shock and after resuscitation

Pooled date of viscoelastic measurements at baseline, in shock and after resuscitation are shown in Table 2. Baseline characteristics of extrinsically and intrinsically stimulated assays matched physiologic human values. Clot firmness in fibrin polymerization assays in swine were higher compared to human values. After surgery and hemorrhage, significant shortening of clotting times in extrinsically and intrinsically stimulated assays were measured by both devices (*p* < 0.01 respectively). Shock and resuscitation were accompanied by reduced clot firmness parameters in ClotPro^®^ and ROTEM^®^. Greatest decrease in clot firmness was found in fibrin polymerization assays (Fib-test and FIB-TEM).

**TABLE 2:** Baseline VHA measurements and changes in shock and resuscitation.

Parameter	Baseline	T2	T4	*p*-value T0 vs. T2	*p*-value T0 vs. T4
	(After anesthesia)	(60 min shock)	(120 min resuscitation)		
ROTEM^®^					
EX CT	42 ± 5	38 ± 5	40 ± 7	0.0007	0.2589
EX A5	64 ± 4	60 ± 5	57 ± 7	0.0008	0.0004
EX A10	70 ± 4	66 ± 4	64 ± 7	0.0011	0.0004
EX MCF	72 ± 4	69 ± 4	67 ± 6	0.0051	0.0031
EX ML	9 ± 3	7 ± 3	7 ± 4	0.0259	0.1768
IN CT	140 ± 25	112 ± 20	111 ± 23	<0.0001	0.0002
IN A5	64 ± 5	60 ± 5	57 ± 7	0.0051	0.0001
IN A10	69 ± 4	65 ± 4	63 ± 6	0.0047	<0.0001
IN MCF	70 ± 4	66 ± 4	64 ± 6	0.005	0.0007
IN ML	15 ± 3	14 ± 4	14 ± 5	0.5432	0.3133
FIB CT	42 ± 4	37 ± 5	41 ± 8	<0.0001	0.3026
FIB A5	41 ± 7	29 ± 6	24 ± 9	<0.0001	<0.0001
FIB A10	46 ± 7	34 ± 5	27 ± 11	<0.0001	<0.0001
FIB MCF	47 ± 7	35 ± 5	29 ± 11	<0.0001	<0.0001
FIB ML	5 ± 3	6 ± 4	9 ± 6	0.1141	0.0122
AP CT	40 ± 4	35 ± 3	39 ± 8	<0.0001	0.4559
AP A5	65 ± 4	60 ± 5	57 ± 7	0.0002	<0.0001
AP A10	71 ± 4	66 ± 5	64 ± 6	0.0001	<0.0001
AP MCF	73 ± 4	68 ± 4	67 ± 6	0.0004	0.0002
AP ML	8 ± 3	7 ± 3	7 ± 5	0.0404	0.4538
ClotPro^®^					
EX CT	55 ± 11	46 ± 10	50 ± 11	0.0022	0.0992
EX A5	63 ± 4	59 ± 4	57 ± 6	0.0014	0.0003
EX A10	68 ± 3	64 ± 3	63 ± 5	0.0003	0.0002
EX MCF	69 ± 4	66 ± 3	65 ± 4	0.0017	0.0007
EX ML	10 ± 2	9 ± 3	9 ± 4	0.74	0.5513
IN CT	126 ± 16	108 ± 14	110 ± 25	0.0001	0.0132
IN A5	59 ± 3	56 ± 4	54 ± 6	0.0026	0.0005
IN A10	63 ± 3	60 ± 3	59 ± 5	0.0028	0.0009
IN MCF	64 ± 3	61 ± 3	60 ± 5	0.001	0.002
IN ML	11 ± 2	12 ± 3	11 ± 4	0.4467	0.7291
FIB CT	57 ± 16	44 ± 11	48 ± 16	0.0016	0.0631
FIB A5	43 ± 9	36 ± 9	31 ± 12	0.0111	0.0003
FIB A10	53 ± 6	45 ± 8	39 ± 13	0.0002	<0.0001
FIB MCF	57 ± 6	50 ± 6	44 ± 13	0.0007	0.0001
FIB ML	7 ± 3	5 ± 3	6 ± 7	0.0605	0.6817
AP CT	44 ± 11	35 ± 9	37 ± 13	0.0018	0.0559
AP A5	61 ± 4	58 ± 4	56 ± 6	0.0044	0.0002
AP A10	67 ± 3	63 ± 4	62 ± 5	0.0006	<0.0001
AP MCF	68 ± 2	65 ± 3	63 ± 4	0.0011	0.0001
AP ML	9 ± 2	9 ± 3	9 ± 4	0.9575	0.9628
TPA CT	42 ± 11	33 ± 10	35 ± 10	0.0013	0.0291
TPA A5	61 ± 3	58 ± 5	56 ± 6	0.0017	0.0003
TPA A10	67 ± 3	63 ± 4	62 ± 5	0.0003	0.0001
TPA MCF	68 ± 2	65 ± 4	63 ± 4	0.0002	0.0002
TPA ML	9 ± 2	9 ± 3	9 ± 5	0.8321	0.8652
NA CT	300 ± 180	192 ± 44	169 ± 56	0.0064	0.0014
NA A5	49 ± 10	53 ± 5	52 ± 7	0.0953	0.1695
NA A10	55 ± 10	58 ± 4	58 ± 6	0.1809	0.2488
NA MCF	58 ± 6	59 ± 4	60 ± 6	0.2181	0.2702
NA ML	13 ± 4	11 ± 3	10 ± 5	0.1575	0.0746

Values are given as mean ± standard deviation. Clot firmness parameter (A5, A10, MCF) were measured in mm, clotting times (CT) in seconds and maximum lysis (ML) in percentage. TPA-test and NA-test were available only for ClotPro^®^. p1: comparison baseline (T0) vs 60 min after shock induction (T2); p2: comparison baseline (T0) vs. 120 min of resuscitation (T4).

### 3.4 Device comparison

#### 3.4.1 Tissue factor stimulated assays

**FIGURE 1:**
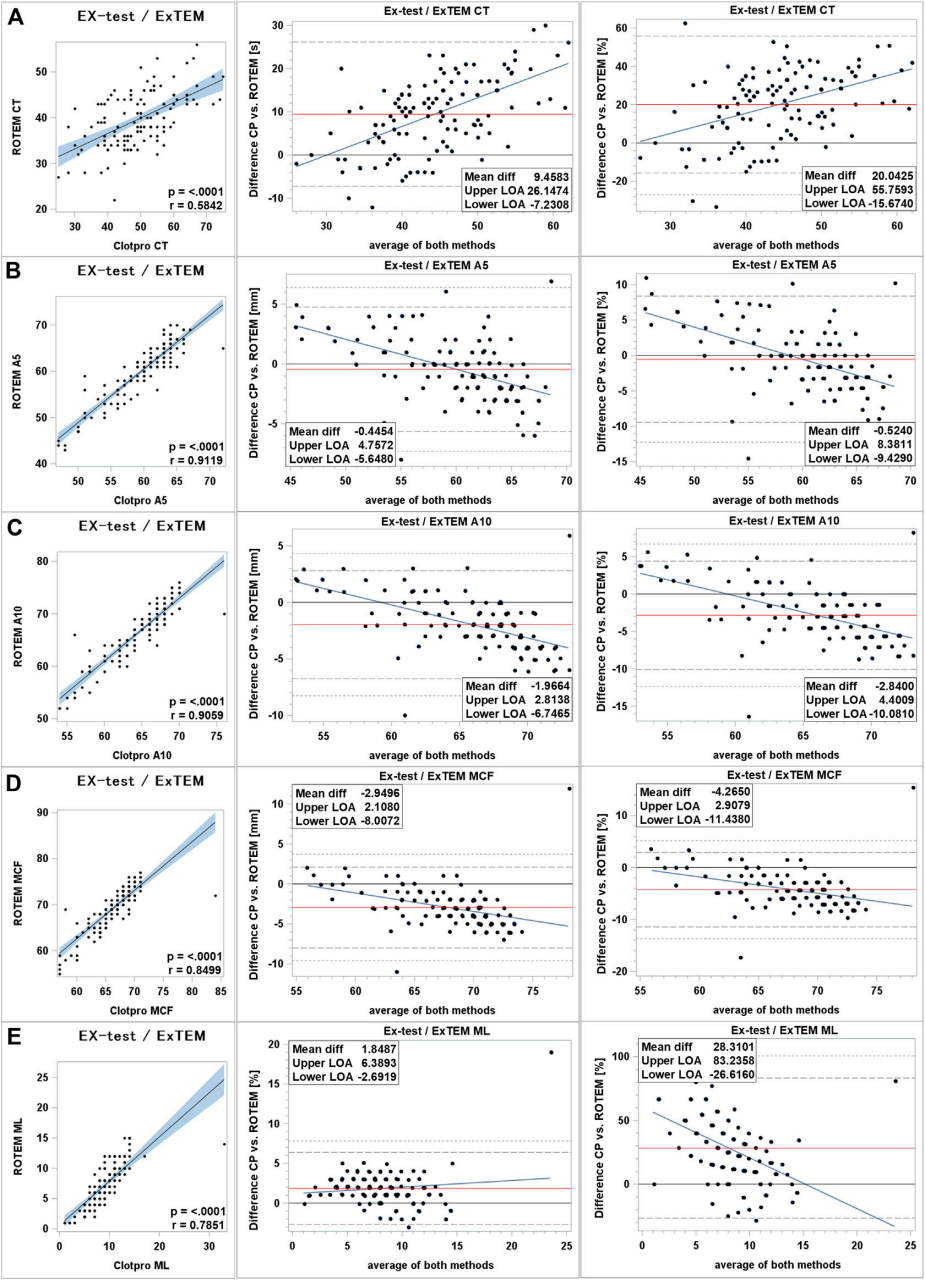
Comparison of extrinsically stimulated assays between ClotPro^®^ and ROTEM^®^. **(A)**: clotting time; **(B)**: clot firmness after 5 min (A5 value); **(C)**: clot firmness after 10 min (A10 value); and **(D)**: maximal clot firmness (MCF value); **(E)**: maximum clot lysis (ML value). On the left: Fit plot with a regression line. The blue area around the regression line represents the 95% confidence limits for the regression line. The *p*-value and Pearson’s correlation coefficient “r” are in the lower right corner. In the middle: Bland–Altman plot of the absolute values. On the right: Bland–Altman plot of percentage values. The solid black line indicates zero difference; the red solid line represents the actual mean bias between the ClotPro^®^ and ROTEM^®^ parameters; The dashed lines represent the 95% limits of acceptance (95% LOA); the dotted line represents the 99% LOA; and the blue line represents the regression line for the difference between ClotPro^®^ and ROTEM^®^. The numeric values of the mean bias and of the lower and upper 95% LOA are given in the bottom right corner. The clotting times are measured in seconds, and the clot firmness is measured in mm amplitude, respectively.

Clot initiation: Tissue factor stimulated assays showed fair correlation between ROTEM^®^ and ClotPro^®^ with Pearson correlation coefficient of 0.58 (*p* < 0.0001). On average ClotPro^®^ values were higher compared to ROTEM^®^ with a mean difference of 9.4 s (+20%). We found wide 95% LOA ranging from −16 to +55%. There was a positive proportional bias increasing at the upper end of measurement range and additional random bias (Figure 1).

Clot firmness: We found very strong correlations between ROTEM^®^ and ClotPro^®^ clot firmness parameters in tissue factor stimulated assays. On average clot firmness parameters were lower in ClotPro^®^ but mean difference was small, ranging from −0.5 mm for A5 to −3 mm for MCF (relative difference −0.5% to −3% respectively). There was a trend showing higher ClotPro^®^ parameters at the bottom range of measurement and lower ClotPro^®^ values at the upper end of measurement range compared to ROTEM^®^. For clot firmness values below 59 mm A10 ClotPro^®^ parameters were higher compared to ROTEM^®^ (Figure 1).

Clot lysis: Clot lysis parameters showed moderate correlation with a correlation coefficient of 0.75 (*p* < 0.0001). Maximum lysis was higher in ClotPro^®^ compared to ROTEM^®^. Absolute mean difference was +1.8% and relative mean difference 28%. We found a wide 95% LOA ranging up to +83% relative difference between devices. Bias increased at the upper range of measurement (Figure 1).

#### 3.4.2 Intrinsically stimulated assays

**FIGURE 2:**
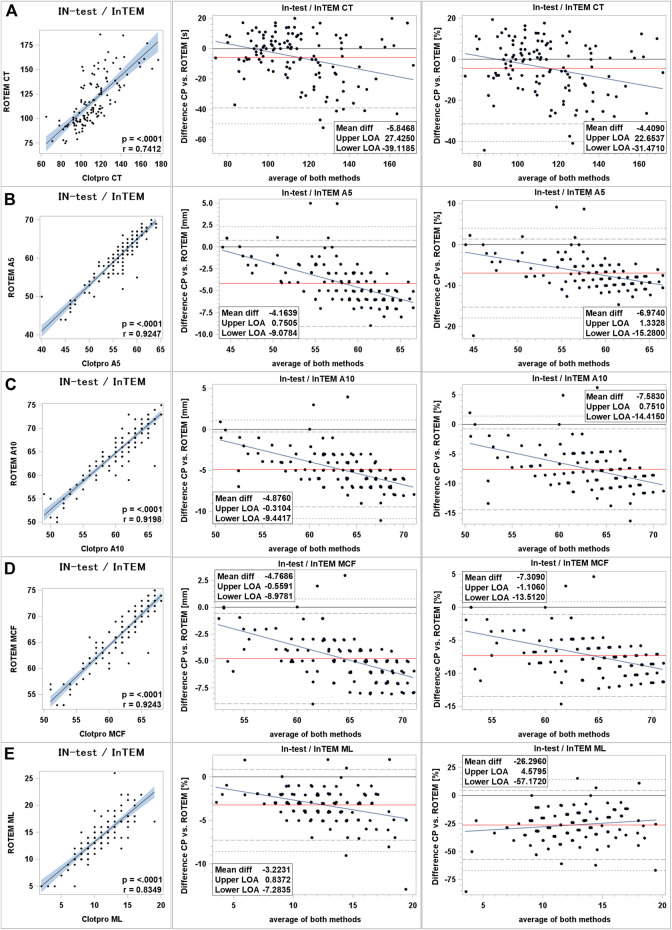
Comparison of intrinsically stimulated assays between ClotPro^®^ and ROTEM^®^. **(A)**: clotting time; **(B)**: clot firmness after 5 min (A5 value); **(C)**: clot firmness after 10 min (A10 value); and **(D)**: maximal clot firmness (MCF value); **(E)**: maximum clot lysis (ML value). On the left: Fit plot with a regression line. The blue area around the regression line represents the 95% confidence limits for the regression line. The *p*-value and Pearson’s correlation coefficient “r” are in the lower right corner. In the middle: Bland–Altman plot of the absolute values. On the right: Bland–Altman plot of percentage values. The solid black line indicates zero difference; the red solid line represents the actual mean bias between the ClotPro^®^ and ROTEM^®^ parameters; The dashed lines represent the 95% limits of acceptance (95% LOA); the dotted line represents the 99% LOA; and the blue line represents the regression line for the difference between ClotPro^®^ and ROTEM^®^. The numeric values of the mean bias and of the lower and upper 95% LOA are given in the bottom right corner. The clotting times are measured in seconds, and the clot firmness is measured in mm amplitude, respectively.

Clot initiation: Data showed moderate correlation between ClotPro^®^ and ROTEM^®^ intrinsically stimulated assays. Correlation coefficient was measured as 0.74 (*p* < 0.0001). Clot initiation was faster in ClotPro^®^ with a mean bias of −6 s (−4%) and differences increased at the upper end of measurement range. 95% LOA was wide ranging from −31% to +22% (Figure 2).

Clot firmness: Clot firmness parameters showed very strong correlations between both devices (correlation coefficients all >0.90). Clot firmness measured by ClotPro^®^ was lower compared to ROTEM^®^. For intrinsically stimulated assays absolute differences ranged from −4 mm (A5 value) to −5 mm (A10 and MCF values). 95% LOA was of moderate span ranging from −15% to +1% relative difference. BA-plots showed that difference between devices become greater at the upper end of measurement range (Figure 2).

Clot lysis: Maximum lysis in intrinsically stimulated assays showed strong correlation between both devices. Absolute mean difference was −3% (−26% relative difference) with a wide 95% LOA (−57% to +4% relative difference). Inter-device difference increased in samples with higher maximum clot lysis (Figure 2).

#### 3.4.3 Fibrin polymerization assays

**FIGURE 3:**
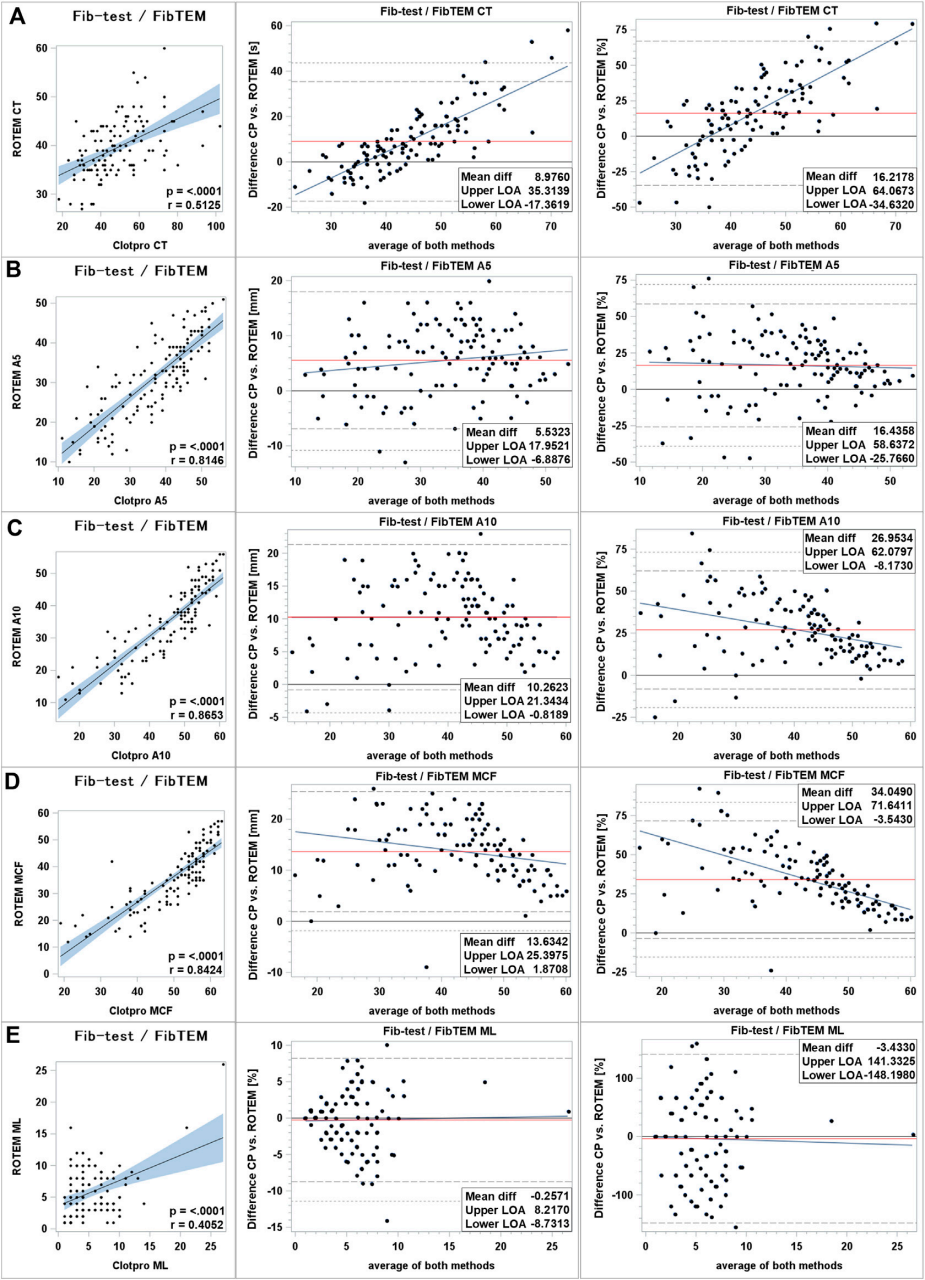
Comparison of fibrin polymerization assays between ClotPro^®^ and ROTEM^®^. **(A)**: clotting time; **(B)**: clot firmness after 5 min (A5 value); **(C)**: clot firmness after 10 min (A10 value); and **(D)**: maximal clot firmness (MCF value); **(E)**: maximum clot lysis (ML value). On the left: Fit plot with a regression line. The blue area around the regression line represents the 95% confidence limits for the regression line. The *p*-value and Pearson’s correlation coefficient “r” are in the lower right corner. In the middle: Bland–Altman plot of the absolute values. On the right: Bland–Altman plot of percentage values. The solid black line indicates zero difference; the red solid line represents the actual mean bias between the ClotPro^®^ and ROTEM^®^ parameters; The dashed lines represent the 95% limits of acceptance (95% LOA); the dotted line represents the 99% LOA; and the blue line represents the regression line for the difference between ClotPro^®^ and ROTEM^®^. The numeric values of the mean bias and of the lower and upper 95% LOA are given in the bottom right corner. The clotting times are measured in seconds, and the clot firmness is measured in mm amplitude, respectively.

Clot initiation: Moderate correlation between ClotPro^®^ and ROTEM^®^ fibrin polymerization assays was found. Correlation coefficient was measured as 0.51 (*p* < 0.0001). Clot initiation was delayed in ClotPro^®^ with a mean bias of +9 s (+16%) and differences increased at the upper end of measurement range. 95% LOA was wide ranging from −35% to +67% (Figure 3).

Clot firmness: Very strong correlation was found for clot firmness parameters in fibrin polymerization assays (Pearson correlation coefficient >0.8). Fib-test clot firmness values were higher compared to ROTEM^®^’s FIB-TEM parameters. Mean difference ranged from +6 mm (A5 value) to +13 mm (MCF value). 95% LOA was wide ranging up to +72% for MCF values. Scatter of inter-device differences increased markedly at the lower end of measurement range while absolute mean difference of A10 values was constant over the whole range of measurement (Figure 3).

Clot lysis: Maximum lysis parameters in fibrin polymerization assays showed fair correlation between both devices. Mean difference was low (−0.25% absolute mean difference; −3% relative mean difference) and differences kept constant over the range of measurement. Scatter increased at the upper end of measurement range. 95% LOA was wide ranging from −148% to +141% relative difference (Figure 3).

#### 3.4.4 Comparison of ClotPro^®^ and ROTEM^®^ parameters over time by treatment group

POC VET measurements from both devices over time and depending on resuscitative treatment were shown in Supplementary Figures S1, S2.

Results show that surgical trauma and heamorrhage leads to a stimulation of the coagulation system represented by shortening of clotting times in extrinsically and intrinsically stimulated assays. Dilution coagulopathy was seen under fluid resuscitation. Dilution coagulopathy was represented by a prolongation of clotting times in extrinsically stimulatte tests and a decrease of clot firmness measured in extrinsically stimulated tests and Fibrin-polymerization assays.

Comparing both devices ClotPro^®^ measurements provide longer clotting times in extrinsically stimulated tests independent from treatment. Further fibrin-polymerization assays showed higher A10 and MCF values in ClotPro^®^ compared to the respective ROTEM^®^ equivalents. The difference was higher under norepinephrine resuscitation compared to control and fluid resuscitation.

### 3.5 Comparison of ClotPro^®^ TPA-test to ClotPro^®^ Ex-Test

**FIGURE 4:**
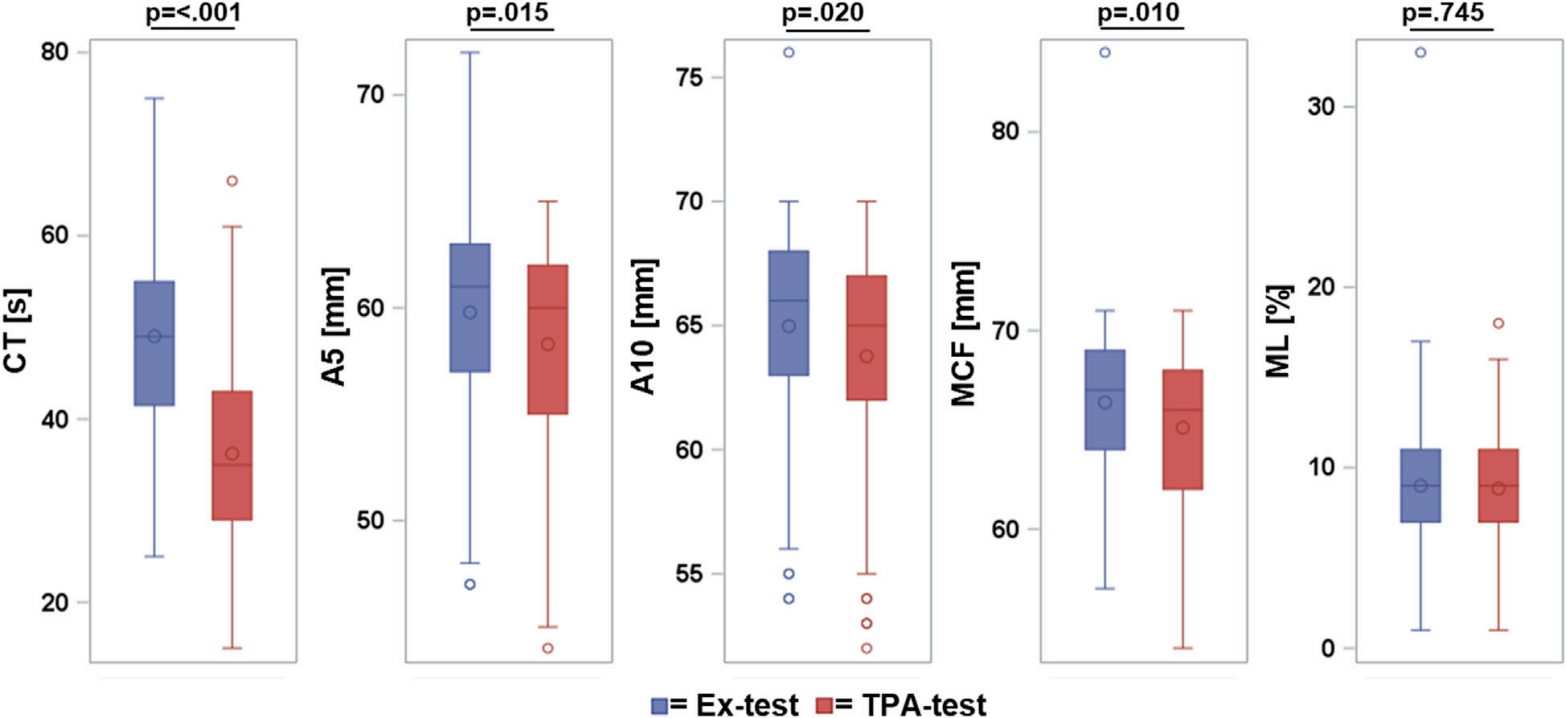
Comparison of ClotPro^®^ Ex-test and TPA-test. Box and whispers plot: box indicates Q1-Q3 inter-quartile range, horizontal line within the box shows median value, marker inside the box displays the mean, whiskers indicates observation nearest to the fence (=1.5 IQR), markers below and above the fences demonstrate observations above and below the fences.

ClotPro^®^ TPA-Test and Ex-Test provide lower values for clot initiation and clot firmness but no significant amplification of clot lysis was found in the presence of tissue-plasminogen activator in TPA-test (Figure 4). Maximum lysis after 60 min of runtime was 8.99% in Ex-test and 8.85% in TPA-test (*p*-value 0.75).

## 4 Discussion

Viscoelastic-based guidance in hemostatic resuscitation provides rapid and holistic view of coagulation and allow customized therapy for the individual patient. Thereby VHAs facilitated a reduction in blood product usage and improved patient outcome and reduces therapy costs. Main limitations of the technique are the need for technical expertise regarding analyzes and interpretation and their yet limited availability.

The primary objective of this study was to assess the applicability of the ClotPro^®^ viscoelastic hemostatic assay in porcine blood samples and to examine the interchangeability of ClotPro^®^ and ROTEM^®^ delta across a broad spectrum of measurements, spanning from physiologic coagulation to coagulopathy. To address this objective, we performed ClotPro^®^ and ROTEM^®^ in 26 pigs (*sus scrofa* domestica) subjected to an established model of coagulopathy induced by major abdominal surgery, severe hemorrhage and resuscitation.

Our findings indicate that ClotPro^®^ is suitable to perform viscoelastic hemostatic measurements in porcine blood samples. Both devices (ClotPro^®^ and ROTEM^®^) provide baseline values for intrinsically and extrinsically stimulated tests comparable to human normal range (Lang et al., 2005; Yoon, 2023). The surgical intervention and shock induction led to the stimulation of the coagulation system, evidenced by a significant shortening of clotting times in extrinsic and intrinsic assays (Table 2; Supplementary Figures S1–S3). Coagulopathy was detected after shock and resuscitation in both devices represented as significantly reduced clot firmness parameters in extrinsic and fibrin polymerization assays (Table 2; Supplementary Figures S1–S3).

In terms of comparability, clot firmness parameters from extrinsically stimulated assays were found to be interchangeable. However, marked differences were observed in intrinsic and extrinsic clotting times, intrinsic clot firmness, and fibrinogen polymerization assay parameters.

Clotting times in extrinsic and intrinsic assays showed only fair to moderate correlation, with wide 95% limits of agreement (LOA) indicating a relative difference of more than 15%. Notably, ClotPro^®^ Ex-test exhibited delayed clot initiation compared to ROTEM^®^. These inter-device differences were even more pronounced in coagulopathic samples. For IN-test/In-TEM CT comparison, mean bias was acceptable with 4% relative difference but random bias was high reflected by a wide 95% LOA.

Clot firmness parameters in tissue-factor-stimulated tests could be used interchangeably within the normal range of measurement, with relative differences between devices at approximately 10%. However, a trend towards higher values in ClotPro^®^ compared to ROTEM^®^ was noted at the lower range of measurement, suggesting that small biases observed under physiological circumstances may increase in severely coagulopathic individuals, potentially leading to reduced sensitivity in detecting coagulopathy when ClotPro^®^ is used compared to ROTEM^®^ delta.

In Fibrin polymerization assays all clot firmness parameters (A5, A10 and MCF) showed clinically relevant differences between devices with higher values measured by ClotPro^®^. Relative differences exceeded 10% in all parameters. The variance in inter-device differences expanded notably at the lower range of measurement, suggesting diminished accuracy. Unfortunately, the determination of whether reduced accuracy occurred in one or both devices could not be ascertained, as no gold standard for actual fibrinogen concentration was measured. Consequently, the study’s results demonstrate the challenge of comparability when assessing Fib-test/FIB-TEM parameters across different devices, particularly at the lower end of the measurement range.

Analyzing VHA measurements in the context of applied treatment revealed that especially in the norepinephrine group a marked difference in Fib-test/FibTEM clot firmness measurements emerged in shock and persists under norepinephrine resuscitation while this was not the case in both other treatment groups. While Fib-test/FibTEM clot firmness decreased in control group and under fluid resuscitation, no further decrease was found under norepinephrine resuscitation. Under resuscitation higher clot firmness values were measured in ClotPro^®^ compared to ROTEM^®^ delta. One potential reason for the preserved Fib-test/FibTEM clot firmness under norepinephrine resuscitation could be the effect of norepinephrine on platelet function. It is shown that catecholamines facilitate platelet activation (Ardlie et al., 1985; Anfossi and Trovati, 1996). Since Fib-test clot firmness parameters were higher in ClotPro^®^ one can hypothesize that the platelet component is more pronounced in ClotPro^®^ Fib-test assay compared to ROTEM^®^ delta’s FibTEM. Another explanation of the observed differences between both devices could be that ClotPro^®^ and ROTEM^®^ delta might detect hypofibrinogenemia with different sensitivity. White et al. saw a profound decrease in fibrinogen concentration in a porcine trauma and hemorrhage model and linked fibrinogen consumption to systemic acidosis (White et al., 2010). Acidosis was most prominent in the norepinephrine group and observed inter-device differences may reflect different sensitivity in detection of severe hypofibrinogenemia. Since the given experiment did not include any gold standard assays for platelet function and actual fibrinogen concentration, further examinations are needed to exactly characterize and explain the effect of catecholamines on fibrin-polymerization assays.

An intriguing finding was the consistently higher values observed in fibrin polymerization assays for both ROTEM^®^ and ClotPro^®^ compared to human data. Despite the reported similarity in fibrinogen concentration between human and swine (Nellenbach et al., 2020), the results demonstrated markedly higher clot firmness parameters in porcine blood samples. Fibrin polymerization assays rely on platelet inhibition to abolish platelet component in clot firmness parameters. Consequently, the measurement should exclusively capture fibrin polymerization, and the resultant clot firmness values are anticipated to exhibit proportionality to fibrinogen concentration. Several studies evaluated ROTEM^®^ FIB-TEM clot firmness in swine. Interestingly all of them showed clot firmness parameters markedly higher than reported in human (Lang et al., 2005; Velik-Salchner et al., 2006; Mauch et al., 2011; Reed et al., 2020). In the FIB-TEM assay Cytochalasin D is used for platelet inhibition. Velik-Salchner et al. found that FIB-TEM MCF in porcine blood samples could be reduced by increasing the cytochalasin D concentrations (Velik-Salchner et al., 2006). The effect was dose dependent without reaching a plateau phase, leading to the hypothesis that Cytochalasin D concentration in FIB-TEM is insufficient to achieve comprehensive platelet inhibition in porcine blood samples. These findings explained higher clot firmness values observed in swine compared to humans.

Until now, no data were available for ClotPro^®^ fibrin polymerization assays in porcine blood samples. These data are of particular interest since ClotPro^®^ Fib-test assay utilizes dual platelet inhibition with cytochalasin D and tirofiban. Due to the dual platelet inhibition, one would expect better platelet inhibition in ClotPro^®^, resulting in reduced platelet component on Fib-test clot firmness parameters. Contrary to expectations, our study demonstrated that dual platelet inhibition did not result in lower clot firmness parameters in ClotPro^®^ compared to ROTEM^®^. In fact, all three clot firmness parameter (A5, A10 and MCF) measured with ClotPro^®^ Fib-test were consistently higher compared to ROTEM^®^ values measured in the same samples. We hypothesed that dual platelet inhibition does not improve platelet inhibition in the ClotPro^®^ fibrin polymerization assay. Our hypothesis is supported by Ciborowski et al. who examined the effect of tirofiban on porcine platelets. They found that tirofiban failed to block tissue-factor induced thrombus formation even in high concentrations. These findings explain the phenomenon that fibrin polimerization assays performed with porcine blood still show platelet component despite dual platelet inhibition in ClotPro^®^ Fib-test. These findings suggest that Cytochalasin D does not sufficiently block platelet activation in fibrin polymerization assays and dual platelet inhibition in ClotPro^®^ Fib-test does not appear to overcome this issue. Therefore clot firmness parameters in porice blood samples were biased by platelet activation and may not correlate sufficiently with actual fibrinogen concentration.

Another relevant question was the applicability of ClotPro^®^ TPA-test in swine. TPA-test is a tissue-factor activated assay wherein 650 ng/mL recombinant tissue plasminogen activator (t-PA) were added to facilitate activation of plasminogen and consequently initiating fibrinolysis. TPA-test aims to measure fibrinolytic potential and monitor antifibrinolytic agents (Bachler et al., 2021; Duque et al., 2021; Groene et al., 2021; Heubner et al., 2022). Given the investigations of tranexamic acid effects in several porcine models of bleeding and hemorrhage (Zentai et al., 2016; Baucom et al., 2022; Lynghaug et al., 2023), it seemed logical to utilize the ClotPro^®^ TPA-test to evaluate fibrinolytic potential and the effects of tranexamic acid in porcine coagulopathy models. Consequently, we explored the applicability of the TPA-test in such a porcine model and found that t-PA in a concentration of 650 ng/mL as used in ClotPro^®^ TPA-test failed to stimulate fibrinolysis in porcine blood samples.

Following a 60-min runtime, no significant difference in maximal clot lysis was observed between ClotPro^®^ TPA-test and Ex-test. These results appear conflicting since pigs were shown before to develop increased fibrinolysis due to elevated t-PA plasma levels (Villeda et al., 1995). However, Korninger and Collen (1981) demonstrated significant interspecies variation in the degree of clot lysis induced by t-PA. These results were confirmed by Yakovlev et al. who revealed that porcine plasminogen is activated by t-PA at a very low rate compared to human plasminogen (Yakovlev et al., 1995). Considering these findings alongside our results, we conclude that TPA-test is not sufficient to study fibrinolytic potential and monitor antifibrinolytic drugs in porcine blood because porcine plasminogen seems not to be activated sufficiently by t-PA concentrations provided in ClotPro^®^ TPA-test.

In summary, our study in a porcine model of surgery, hemorrhage, and resuscitation highlights the applicability of ClotPro^®^ viscoelastic hemostatic assays in porcine blood samples. However, caution is advised in interpreting results, as device-specific normal values exist, and interchangeability is limited to certain parameters. Particularly for in- and extrinsic clotting times and clot firmness in fibrin polymerization assays clinically relevant differences exist.

Recent head-to-head comparisons between ClotPro^®^ and ROTEM^®^ in human demonstrated notable differences in clotting times in extrinsically and intrinsically stimulated tests, interchangeability of clot firmness in tissue-factor activated tests, and higher clot firmness values in fibrin polymerization assays for ClotPro^®^ compared to ROTEM^®^ (Infanger et al., 2021; Yoshii et al., 2022; Gruneberg et al., 2024). Our study confirmed the presence of all these device-specific differences in porcine blood samples and revealed that dual platelet inhibition in ClotPro^®^ has no benefit compared to singular platelet inhibition in ROTEM^®^. Additionally, we found that ClotPro^®^ TPA-test is not applicable in swine for studying fibrinolytic potential and monitoring antifibrinolytic agents.

### 4.1 Limitations

The present study is subject to certain limitations. Owing to slight differences in the coagulation systems of various species, the results obtained herein cannot be extrapolated to animal models of different species. As Frith et al. (2012) mentioned in their literature review in 2011, pigs show a hypercoagulable state compared to human and sensitivity of human coagulation assays in detection of coagulopathy may leck when animal samples are used. Additional limitations stem from the fact that only viscoelastic hemostatic measurements were conducted, lacking standard coagulation laboratory data and individual factor concentration measurements as a gold-standard reference. Consequently, determining which of the two tested devices better aligns with the true circumstances was not feasible. To address this question, further studies are required, simultaneously conducting viscoelastic hemostatic assays (VHA) in parallel with other established coagulation tests.

### 4.2 Conclusion

In a porcine model of surgery, hemorrhage and resuscitation, containing 26 pigs (*sus scrofa* domestica) we examined the applicability of the ClotPro^®^ viscoelastic hemostatic assay and evaluate device specific differences and limitations in comparison to ROTEM^®^ delta.

The results demonstrated that ClotPro^®^ is suitable for performing viscoelastic hemostatic measurements in porcine blood samples. However, caution is warranted in interpreting the findings as device-specific differences exist, particularly in in- and extrinsic clotting times and clot firmness in fibrin polymerization assays. Only Ex-test and In-test clot firmness parameters could be used interchangeably with their ROTEM^®^ delta equivalents. We found that dual platelet inhibition in ClotPro^®^ has no additional effect over singular platelet inhibition in ROTEM^®^ delta so that the problem of insufficient platelet inhibition in ROTEM^®^ FIB-TEM does not seem to be overcome in ClotPro^®^.

The study also evaluated the ClotPro^®^ TPA-test, revealing its inapplicability in swine for studying fibrinolytic potential and monitoring antifibrinolytic agents. The TPA-test failed to stimulate fibrinolysis in porcine blood samples, highlighting species-specific variations in the activation of porcine plasminogen by tissue plasminogen activator.

This study contributes to the understanding of ClotPro^®^’s performance in porcine blood samples. Further research, incorporating additional coagulation tests, is recommended to enhance the assessment of device alignment with true circumstances in porcine blood samples.

## Data Availability

The original contributions presented in the study are included in the article/Supplementary Material, further inquiries can be directed to the corresponding author.
