# Supplementary figures and images for: Comparison of two viscoelastic testing devices in a porcine model of surgery, hemorrhage and resuscitation
Source: Front Bioeng Biotechnol. 2024 Aug 13;12:1417847. doi: 10.3389/fbioe.2024.1417847 (PMC11347288; doi:10.3389/fbioe.2024.1417847)

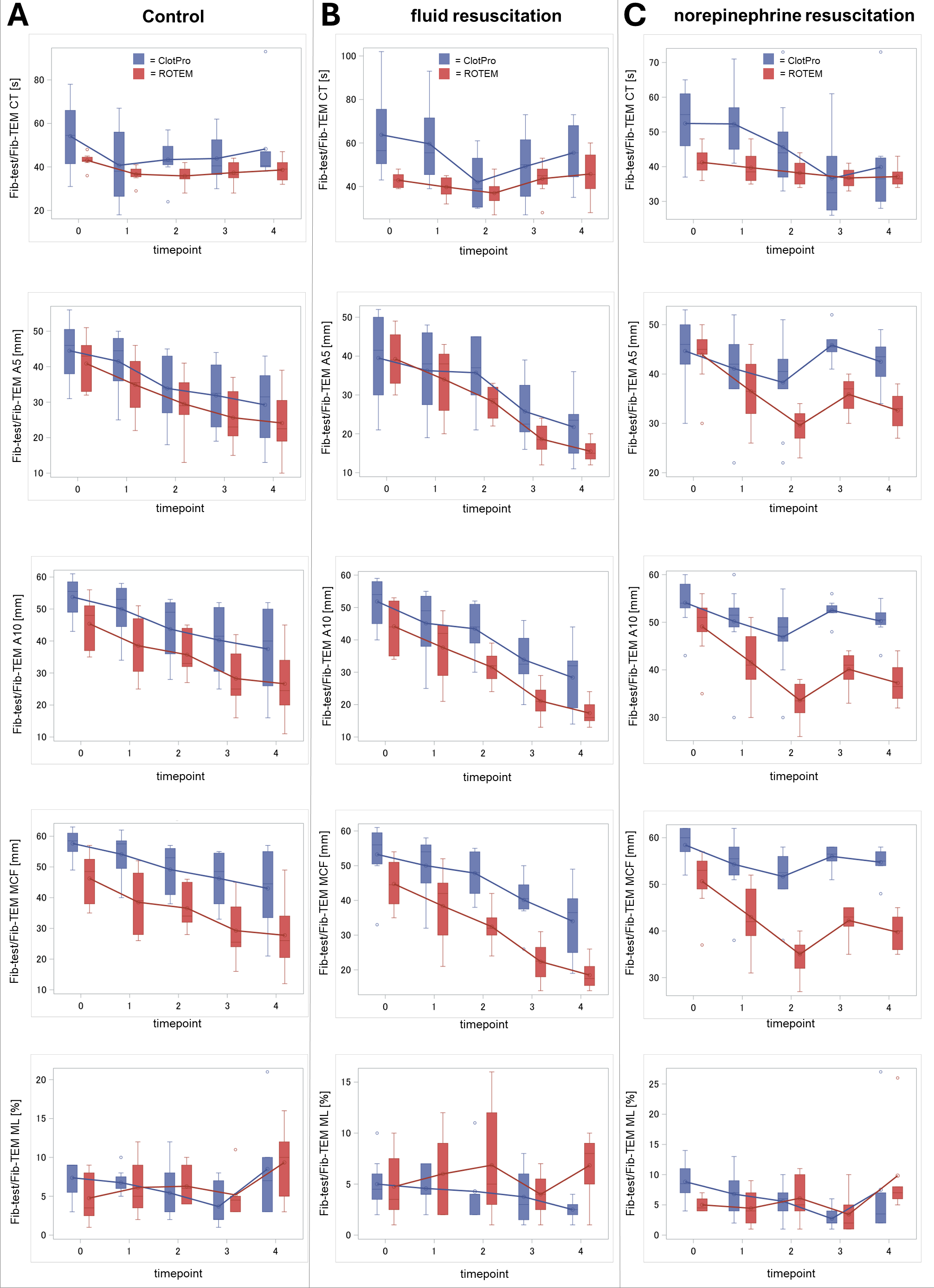

Supplement: Supplementary file 1 [file Image3.TIF]

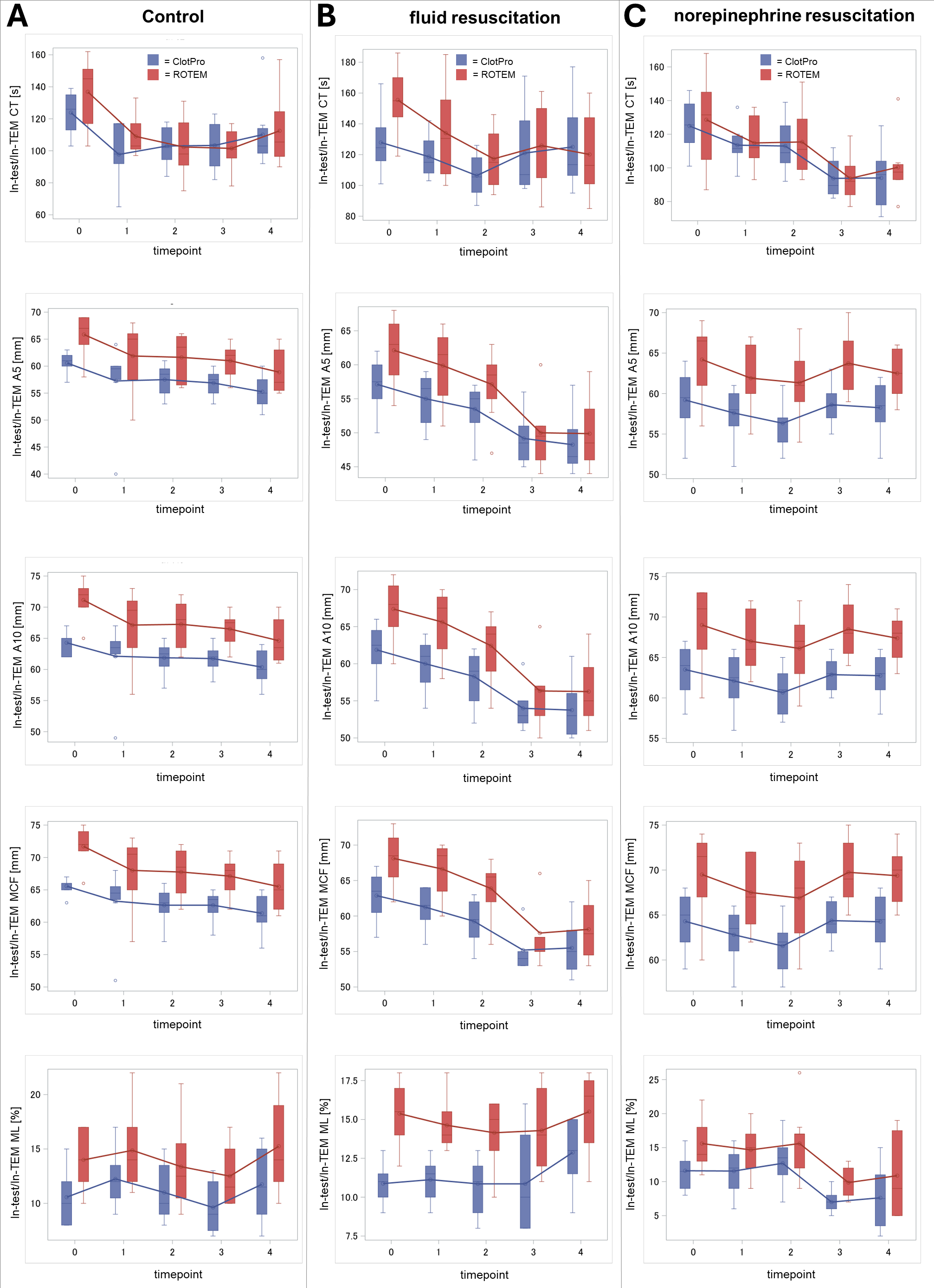

Supplement: Supplementary file 2 [file Image2.TIF]

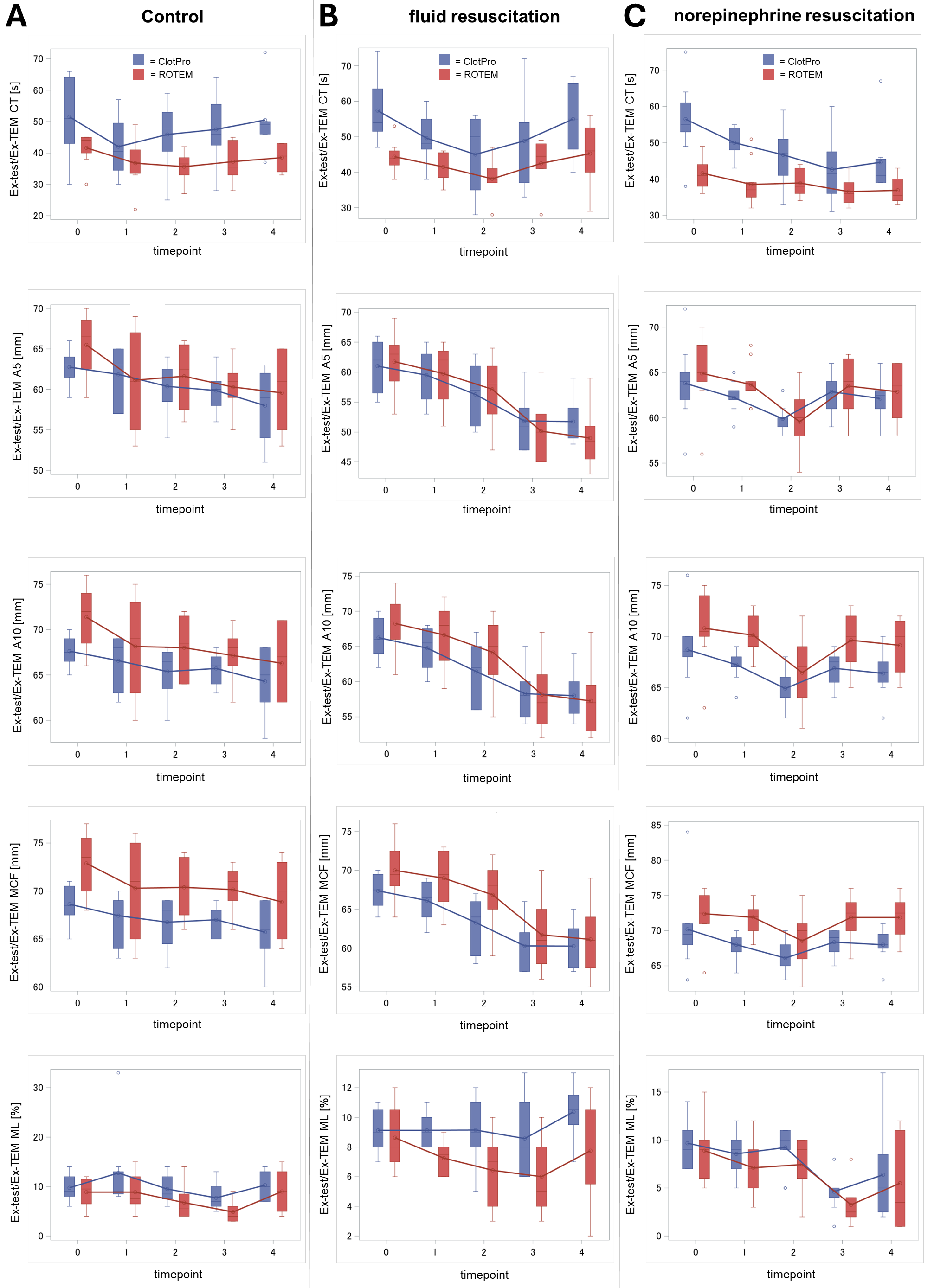

Supplement: Supplementary file 3 [file Image1.TIF]
